# Single cobalt atoms with unconventional dynamic coordination mechanism for selective ammonia sensor

**DOI:** 10.1093/nsr/nwaf031

**Published:** 2025-02-04

**Authors:** Yuejiao Li, Yaguang Li, Yushu Shi, Jianmei Gao, Jianmin Lu, Chao Wang, Junyu Chang, Zhenming Wang, Yangyue Yang, Bing Yang, Liang Feng, Qiang Fu, Xinhe Bao, Zhong-Shuai Wu

**Affiliations:** State Key Laboratory of Catalysis, Dalian Institute of Chemical Physics, Chinese Academy of Sciences, Dalian 116023, China; State Key Laboratory of Catalysis, Dalian Institute of Chemical Physics, Chinese Academy of Sciences, Dalian 116023, China; Research Center for Solar Driven Carbon Neutrality, Engineering Research Center of Zero-Carbon Energy Buildings and Measurement Techniques, Ministry of Education, The College of Physics Science and Technology, Institute of Life Science and Green Development, Hebei University, Baoding 071002, China; Department of Instrumentation and Analytical Chemistry, CAS Key Laboratory of Separation Science for Analytical Chemistry, Dalian Institute of Chemical Physics, Chinese Academy of Sciences, Dalian 116023, China; University of Chinese Academy of Sciences, Beijing 100049, China; Department of Instrumentation and Analytical Chemistry, CAS Key Laboratory of Separation Science for Analytical Chemistry, Dalian Institute of Chemical Physics, Chinese Academy of Sciences, Dalian 116023, China; State Key Laboratory of Catalysis, Dalian Institute of Chemical Physics, Chinese Academy of Sciences, Dalian 116023, China; State Key Laboratory of Catalysis, Dalian Institute of Chemical Physics, Chinese Academy of Sciences, Dalian 116023, China; Department of Instrumentation and Analytical Chemistry, CAS Key Laboratory of Separation Science for Analytical Chemistry, Dalian Institute of Chemical Physics, Chinese Academy of Sciences, Dalian 116023, China; Department of Instrumentation and Analytical Chemistry, CAS Key Laboratory of Separation Science for Analytical Chemistry, Dalian Institute of Chemical Physics, Chinese Academy of Sciences, Dalian 116023, China; Environmental Research Institute, Shandong University, Qingdao 266237, China; Department of Instrumentation and Analytical Chemistry, CAS Key Laboratory of Separation Science for Analytical Chemistry, Dalian Institute of Chemical Physics, Chinese Academy of Sciences, Dalian 116023, China; University of Chinese Academy of Sciences, Beijing 100049, China; Dalian National Laboratory for Clean Energy, Chinese Academy of Sciences, Dalian 116023, China; Department of Instrumentation and Analytical Chemistry, CAS Key Laboratory of Separation Science for Analytical Chemistry, Dalian Institute of Chemical Physics, Chinese Academy of Sciences, Dalian 116023, China; State Key Laboratory of Catalysis, Dalian Institute of Chemical Physics, Chinese Academy of Sciences, Dalian 116023, China; State Key Laboratory of Catalysis, Dalian Institute of Chemical Physics, Chinese Academy of Sciences, Dalian 116023, China; State Key Laboratory of Catalysis, Dalian Institute of Chemical Physics, Chinese Academy of Sciences, Dalian 116023, China; Dalian National Laboratory for Clean Energy, Chinese Academy of Sciences, Dalian 116023, China

**Keywords:** single atom, graphene, gas sensor, ultra-selective, coordination chemistry

## Abstract

Developing gas sensors that can simultaneously achieve high sensitivity and selectivity for the detection of a single-type gas remains a significant challenge. Herein we demonstrate cobalt (Co) single atoms with an unconventional dynamically changing coordination structure that could be used as NH_3_-sensing material with superior sensitivity and selectivity. According to the steric effect of 2-methylimidazole (2MI) molecules and carbonyl groups on graphene, the Co single atom is evolved into a bidentate coordinated structure (Co-2MI-G). *In-situ* characterization and theoretical simulation reveal that the sensing mechanism of Co-2MI-G is the specific chemical adsorption between unsaturated coordinated Co single atoms and NH_3_ molecules, causing a reversible switching of coordination number from 2 to 4, a valence state transfer from Co^2+^ to Co^3+^ of Co single atoms, and a band-gap width from 0.14 eV to 0.50 eV. Consequently, the Co-2MI-G-based gas sensor presents a sensing response of 67.598% for 1 ppm NH_3_ and a limit of detection of 2.67 ppb, at least 1.8 times higher than that of state-of-the-art NH_3_ sensors, together with robust stability and reproducibility. This work provides an innovative perspective on utilizing single atoms for ultra-selective gas sensing by coordination regulation.

## Teaser

This work presents cobalt (Co) single atoms, with a dynamically changing coordination structure, that achieve superior sensitivity and selectivity for NH_3_ gas sensing.

## INTRODUCTION

Gas-sensing technology is very important for many widespread applications in industrial production, automotive industry, medical supervision, indoor air quality management and environmental monitoring [1–4]. To date, various semiconductor materials [5–8] and ion-conducting hydrogels have been developed for practical gas sensing [9–12]. However, the gas adsorption process usually has limitations when it comes to selectively detecting single types of gas due to the non-specific interactions between gas molecules and sensing materials [13–15]. In particular, achieving highly sensitive and selective detection of single-type NH_3_ gas at room temperature remains a major challenge. So far, Cu_3_(HHTP)_2_ thin-film gas sensors have exhibited the best resistance response of 50% to 10 ppm NH_3_, with a response ratio ranging from 1.25 to 2.8 for 10 ppm NH_3_ and 100 ppm of volatile organic compounds (VOCs) [16]. However, they still cannot selectively distinguish single-type NH_3_ gas from complex interfering gases [17,18]. Therefore, it is necessary to design highly sensitive and selective sensing materials for single-type gas detection.

Single-atom catalysts, which enable the rational use of metal resource and achieve atomic economy, feature isolated metal atoms anchored on solid supports [19–21] to maximize metal dispersion and atom-utilization efficiency. Single metal atoms, with unique electronic structures and specific volume-to-surface properties, differ from conventional metal-based nanoparticles and exhibit distinct physical and chemical properties with remarkable performance [22,23]. In particular, recent studies have demonstrated the unparalleled advantages of single-metal atoms in the catalysis conversion of gas molecules, such as CH_4_ [24], CO_2_ [25] and NH_3_ [26]. Inspired by this, using single atoms as sensing sites to construct a gas sensor would be a better solution [27,28].

Herein, we report a novel gas sensor based on cobalt (Co) single-metal atoms with an organic-ligand-coordinated structure (Co-2MI-G). Different from previous Co single atoms with a quadridentate coordinated structure [29,30], the steric effect of organic ligands was utilized to create bidentate-coordinated Co single atoms, enabling strong selective interaction with NH_3_ molecules. Additionally, the coordination number of the Co single atom could dynamically change during the NH_3_ adsorption/desorption process, resulting in high sensitivity and unprecedented selectivity in NH_3_ sensing, superior to state-of-the-art gas sensors. Moreover, the unconventional sensing mechanism based on coordination chemistry was verified, explaining how low-coordinated Co single atoms were specific adsorption sites for NH_3_ molecules.

## RESULTS AND DISCUSSION

### Synthesis and characterization of Co-2MI-G

**Figure 1.**
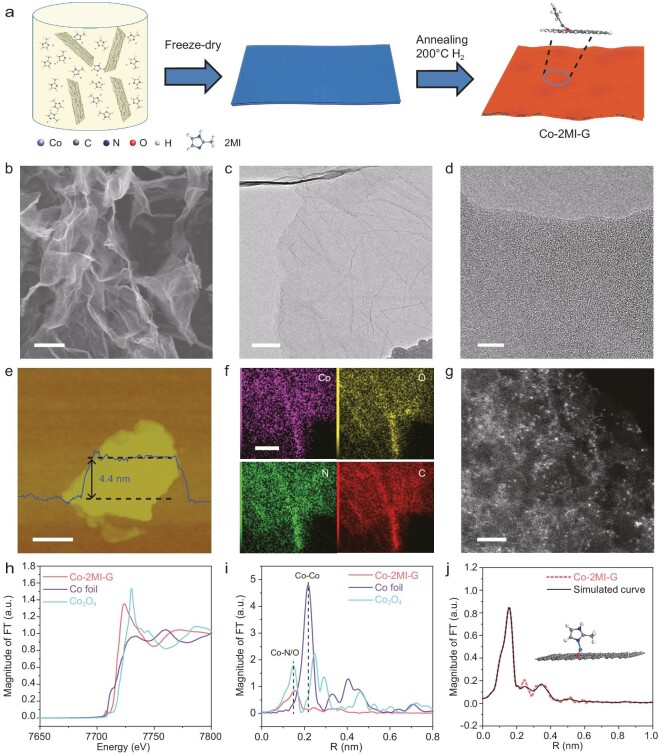
Synthesis and characterizations of Co-2MI-G. (a) The scheme of preparing Co-2MI-G. Cyan, gray, blue, red and pale spheres are representative of the Co, C, N, O and H atoms, respectively. (b) SEM, (c, d) TEM and (e) AFM images of Co-2MI-G. (f) Element mapping for Co, O, N and C of Co-2MI-G. (g) HAADF-STEM image of Co-2MI-G. (h) XANES and (i) FT-EXAFS spectra at the Co *K*-edge for Co-2MI-G, metallic Co foil and Co_3_O_4_. (j) FT-EXAFS curves of the proposed structure (solid line) and the measured Co-2MI-G (dotted line). Inset is the proposed model of Co-2MI-G architecture. The scale bars in (b–g) are 1 µm, 100 nm, 10 nm, 200 nm, 200 nm and 2 nm, respectively.

The preparation process of Co-2MI-G is schematically depicted in Fig. 1a. First, the oxygen-containing groups on graphene oxide (GO) were used to adsorb and anchor Co^2+^ ions, thus forming evenly dispersed metal ion sites on the surface of GO [31]. Meanwhile, 2-methylimidazole (2MI) was dissolved in this solution to coordinate with the Co^2+^ cations. Finally, Co-2MI-G was obtained after annealing at 200°C under H_2_ atmosphere. X-ray diffraction (XRD) pattern and Raman spectrum validated the existence of slightly reduced graphene and confirmed no formation of Co complex aggregates (Fig. S1) [32–34]. Scanning electron microscopy (SEM, Fig. 1b) and transmission electron microscopy (TEM, Fig. 1c) images presented 2D graphene-like morphology of Co-2MI-G, and further confirmed no aggregates on the surface of graphene (Fig. 1d). Atomic force microscopy (AFM) revealed a uniform thickness of ∼4.4 nm for Co-2MI-G (Fig. 1e), and the specific surface area of Co-2MI-G was 503 m^2^ g^−1^, providing abundant sites for gas adsorption (Fig. S2). Furthermore, the homogeneous distribution of Co, O, N and C in the Co-2MI-G was confirmed by elemental mapping analysis (Fig. 1f). An aberration-corrected high-angle annular dark-field scanning transmission electron microscopy (HAADF-STEM) was employed to detect Co species (Fig. 1g), observing single-atom forms of Co species dispersed in Co-2MI-G. X-ray absorption near-edge structure (XANES) determined that the absorption edge energy (*E_0_* value) of Co-2MI-G, metallic Co foil and Co_3_O_4_ was 7.713, 7.707 and 7.718 KeV respectively. It was indicated that the oxidation state of Co in Co-2MI-G was higher than that in metallic Co foil, but lower than that in Co_3_O_4_ (Fig. 1h). Fourier transformed-extended X-ray absorption fine structure (FT-EXAFS) showed only Co-O/N scattering located at 1.7 Å for Co-2MI-G (Fig. 1i) without any presence of Co-Co metal bond, highlighting the single atomic state of Co-2MI-G. In addition, it was verified from the fitting of FT-EXAFS that the coordination number of Co single atoms in Co-2MI-G was 2 (Table S1), lower than the previously reported Co single atoms with quadridentate coordinated structure [33,35,36]. Therefore, low-coordinated Co single atoms using the organic ligands of 2MI were successfully synthesized. Further, by analyzing the elements of C, N, O in graphene and 2MI ligands in Co-2MI-G (Figs S3 and S4) as well as the C/N/O/Co ratio (Fig. S5), we constructed three representative coordination structures of Co single atoms, including 2MI-Co-2MI, G-O-Co-O-G and G-O-Co-2MI (Fig. S6). Based on the first principal calculation, the only rational structure was the G-O-Co-2MI in terms of energy stability. In this structure, one Co single atom was stably bonded to carbonyl oxygen from graphene and nitrogen from 2MI (inset of Fig. 1j), which is in good agreement with both experimental and simulation FT-EXAFS results for Co-2MI-G (Fig. 1j). The Co-2MI-G has NH_3_ adsorption capacity with a NH_3_ adsorption quantity of 98 mg g^−1^ at room temperature (Fig. S7).

### NH_3_ adsorption/desorption excited coordination evolution

**Figure 2.**
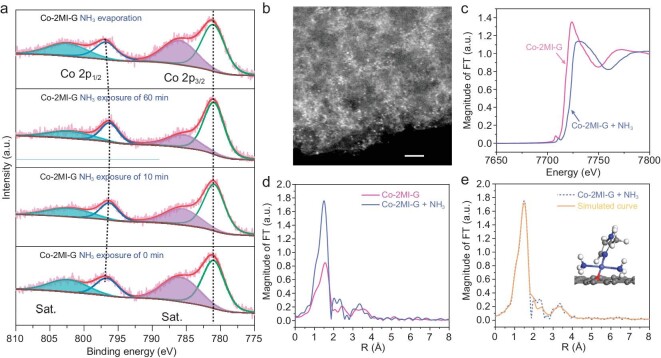
Structural coordination evolution of Co-2MI-G during the NH_3_-sensing process. (a) Quasi *in-situ* Co 2p XPS spectra of Co-2MI-G before and after exposure to NH_3_ for 10 min and 60 min, followed by NH_3_ evaporation. (b) HAADF-STEM image of Co-2MI-G after NH_3_ sensing. (c) XANES and (d) FT-EXAFS spectra at the Co *K*-edge of Co-2MI-G before and after the NH_3_-sensing process. (e) FT-EXAFS curves of the proposed structure (solid line) and the measured Co-2MI-G (dotted line) after the NH_3_-sensing process. Inset is the proposed model of Co-2MI-G architecture after the NH_3_-sensing process. The scale bar in (b) is 2 nm.

The metastable coordinated Co single atoms in Co-2MI-G show a special coordination evolution in the presence of ammonia. To illustrate the inherent evolution mechanism, a series of quasi *in-situ* characterization techniques were carried out to assess the state change of Co single atoms before and after NH_3_ gas exposure. Quasi *in-situ* X-ray photoelectron spectroscopy (XPS) revealed the pristine Co-2MI-G with binding energies of 781.0 eV for Co 2p_3/2_ and 796.6 eV for Co 2p_1/2_, accompanied by a strong shake-up satellite, a typical feature of Co^2+^ (Fig. 2a) [37]. By introducing NH_3_ for 60 min, the 2p_1/2_–2p_3/2_ separation decreased gradually from 15.6 eV to 15.2 eV, while the intensity of the satellite peak decreased significantly, which can be attributed to the conversion of Co^2+^ to Co^3+^ [38,39]. Thus, electron paramagnetic resonance (EPR) spectra were provided to support the hypothesis regarding the change of Co valence in the NH_3_ sensor. The EPR spectrum of pristine Co-2MI-G revealed a broad symmetric line shape without an obvious signal (Fig. S8). Upon exposure to NH_3_ for 60 min, a new sharp signal with *g* = 2.003 was observed, indicative of the valence state transfer of Co^2+^ into Co^3+^ [40]. Further, as illustrated by the HAADF-STEM image, the Co atoms in Co-2MI-G were still maintained in single atomic form after NH_3_ gas exposure (Fig. 2b). As shown in Fig. 2c, XANES confirmed that the *E*_0_ value of Co-2MI-G shifted from 7.713 KeV to 7.721 KeV after NH_3_ exposure [41]. More importantly, FT-EXAFS indicated that the nearest neighbor coordination number of Co in Co-2MI-G was 4 after NH_3_ exposure (Fig. 2d and Table S1), which was significantly higher than the coordination number of 2 in the pristine Co-2MI-G, originating from NH_3_ coordination. Based on this adsorption model, the stable atomic structure of Co-2MI-G bonding with two NH_3_ molecules was constructed (inset of Fig. 2e), and perfectly fitted to the experimental FT-EXAFS result of Co-2MI-G after NH_3_ exposure.

### NH_3_-sensing performance of Co-2MI-G

After finding that the adsorption of NH_3_ will affect the overall coordination environment of Co-2MI-G, we envision using Co-2MI-G as a NH_3_-sensing material.

The effect of Co-2MI-G dosage on sensing performance was investigated first. It was validated that a loading mass of 10 mg was better (Figs S9–S11), indicative of the effectiveness of Co-2MI-G. The resistances of the sensors based on various materials are shown in Fig. S12, in which the baseline resistance changes as expected. Specifically, the thickness of the sensing film is between 42.7 and 53.2 μm, accompanied by a porous structure (Fig. S13), which is conducive to the mass transfer dynamics in the gas sensing process.

**Figure 3.**
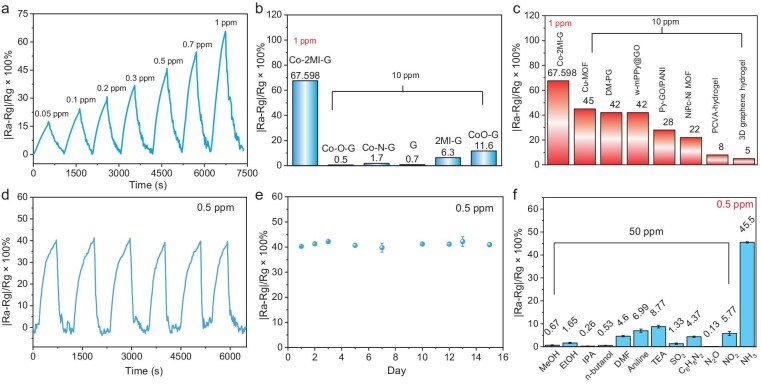
The NH_3_-sensing performance of Co-2MI-G. (a) Resistance response curves of the Co-2MI-G-based sensor towards NH_3_ from 0.05 to 1 ppm. (b) Sensing performance comparison with NH_3_ between Co-2MI-G (1 ppm) and Co-O-G (10 ppm), Co-N-G (10 ppm), G (10 ppm), 2MI-G (10 ppm) and CoO-G (10 ppm). (c) Sensing behavior comparison of Co-2MI-G and other reported NH_3_-sensitive materials. (d) Real-time dynamic response curve upon 0.5 ppm NH_3_ for six cycles. (e) Continuous repeatability under 0.5 ppm NH_3_ of the Co-2MI-G-based sensor. (f) Selectivity of Co-2MI-G to 1 ppm NH_3_ and 50 ppm of other VOCs gases.

Figure 3a shows the typical response–recovery curve of Co-2MI-G to NH_3_ with different concentrations from 0.05 to 1 ppm, exhibiting a dramatically increased resistance response upon exposure to NH_3_. Through calculation, there was a perfect linear relationship between resistance response (*|R_a_-R_g_|/R_g_ ×* 100%) and concentration of NH_3_, with a superior regression coefficient (*R*^2^ = 0.9936) in the range of 0.05 to 1 ppm (Fig. S11). Notably, the sensor exhibits an excellent gas-sensing response to trace NH_3_ (0.01 ppm-17.426%), suggesting the high sensitivity of the Co-2MI-G-based sensor. To characterize the sensing behavior of Co-2MI-G, a series of samples were synthesized for comparison, including Co single atoms bonded to oxygen atoms/graphene (Co-O-G, Fig. S14), Co single atoms bonded to nitrogen atoms/graphene (Co-N-G, Fig. S15), pure graphene (G, Fig. S16), pure 2MI decorated graphene (2MI-G, Fig. S17) and CoO nanoparticles decorated on graphene (CoO-G, Fig. S18). It was demonstrated that Co-2MI-G had the best response towards 1 ppm NH_3_ (67.598%, Fig. 3b), superior to the responses of Co-O-G (0.5%, Fig. S19), Co-N-G (1.7%, Fig. S20), G (0.7%, Fig. S21), 2MI-G (6.3%, Fig. S22), CoO-G (11.6%, Fig. S23), Co-2MI-G-1 (26.9%, Fig. S9) and Co-2MI-G-3 (12.1%, Fig. S10). Moreover, this excellent response of Co-2MI-G towards 1 ppm NH_3_ was significantly superior to other advanced NH_3_ sensors, as summarized in Fig. 3c and Table S2.

It is well established that the kinetics of gas sensing at ambient temperature is typically slow, which is attributed to the adsorption and desorption processes of the gas under consideration. The response and recovery times of the sensor under investigation are illustrated in Fig. S24. It can be observed that the response and recovery times are 6.07 and 5.10 minutes, respectively. Notably, the recovery time of the Co-2MI-G-based sensor is broadly better than that of the reported sensing materials, indicating excellent cyclicity (Table S3).

To evaluate the recyclability of the sensor, we examined the Co-2MI-G-based sensor over six cycles in sequence with 0.5 ppm of NH_3_ (Fig. 3d). The response of the sensor remained almost unchanged (∼90%) even after the sixth cycle, indicating robust stability. Moreover, the Co-2MI-G-based sensor exhibited negligible variations in resistance during periodic exposure to 0.5 ppm of NH_3_ for 15 days, demonstrating extraordinary reproducibility (Fig. 3e). Selectivity represents a crucial parameter in the specific recognition of target gases. To further verify the excellent selectivity of the NH_3_ sensor, the response of the Co-2MI-G-based sensor towards 50 ppm typical VOCs showed a prominent anti-interference performance (Fig. 3f). The responses towards 50 ppm VOCs were all 0.13%∼8.77%, much lower than 45.5% for 0.5 ppm NH_3_ using Co-2MI-G. Notably, a record selectivity of 5.18∼350 was achieved for Co-2MI-G for 0.5 ppm NH_3_/50 ppm VOCs, which was much higher than the reported outstanding selective values (1.25∼2.8) [16,27,28,42–44]. Therefore, it is demonstrated that Co-2MI-G shows the excellent selective sensing performance for NH_3_.

To further verify the selective response of the sensor to specific substances, we selected a number of VOCs containing the element nitrogen as representatives of organic amines and nitrogen oxides (NO_x_). It is notable that the sensor demonstrates a discernible response to amines compared to alcohols, aldehydes and sulfur-containing compounds (Fig. 3f, Fig. S25). It is implied that the nitrogen present in NH_3_ and organic amines exhibits analogous coordination interactions with the Co single atom within Co-2MI-G. However, the response values exhibit variation due to additional factors such as the capacity of the group to gain or lose electrons and steric hindrance.

Sensing tests were also carried out under different relative humidities (RHs) from 25% to 90% to validate the anti-humidity property (Figs S26 and S27), since anti-humidity is one of the important indicators of the sensor in practical application scenarios. The sensor response tended to first increase and then decrease with the increase in humidity. This was attributed to the fact that the presence of H_2_O in relatively high humidity conditions increases the solubility of NH_3_ in H_2_O, thereby facilitating the trace enrichment of NH_3_. However, excessively high humidity results in a significant occupation of adsorption and reaction sites on the material surface, which ultimately leads to a decline in sensing performance. Furthermore, significant variations in sensor resistance under elevated humidity conditions (Fig. S28) can also substantiate the competitive adsorption of H_2_O and NH_3_ on the sensing material surface. Apart from relative humidity, the effect of temperature on sensing is a critical factor, especially for semiconductor resistive gas sensors. It was found that the sensor response value decreased and the response recovery time increased in accordance with the expected relationship, which was attributed to the rapid diffusion of gas molecules and the instability of the sensing process at high temperatures (Fig. S29).

### Theoretical simulation

**Figure 4.**
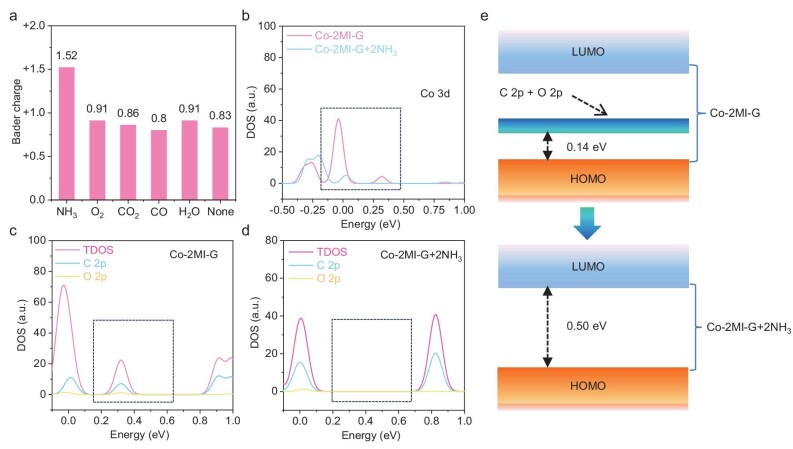
First principle calculations to simulate the NH_3_ chemisorption of Co single atoms in Co-2MI-G for a gas sensor. (a) The Bader charge of Co single atoms in Co-2MI-G after adsorbing different gases. (b) The Co 3d partial DOS of Co-2MI-G and Co-2MI-G + 2NH_3_. (c, d) The total DOS, C 2p partial DOS, O 2p partial DOS of Co-2MI-G and Co-2MI-G + 2NH_3_. (e) Demonstration of frontier molecular orbital structures of Co-2MI-G and Co-2MI-G + 2NH_3_.

Density functional theory (DFT) was then utilized to simulate the NH_3_ chemisorption of Co single atoms in Co-2MI-G. First, we calculated the Bader charge of Co single atoms in Co-2MI-G after adsorbing typical gases used in sensing experiments, including NH_3_, O_2_, CO_2_, CO and H_2_O (Fig. 4a). The simulation showed that the Bader charge of Co single atoms in Co-2MI-G was +1.52, +0.91, +0.86, +0.8 and +0.91 after adsorbing NH_3_, O_2_, CO_2_, CO and H_2_O, respectively, different from the +0.83 Bader charge of Co single atoms in pristine Co-2MI-G (Fig. 4a). Moreover, by calculating the frontier molecular orbitals of Co-2MI-G before and after NH_3_ adsorption (Fig. 4b), the structural change revealed the extraction of Co 3d electrons near the Fermi surface. This extraction of 3d electrons of Co single atoms brought about a significant change in the conductivity of Co-2MI-G. The band gap of Co-2MI-G is the hybridization of Co single atoms, 2MI and graphene (Fig. S30). In the total density of states (DOS) diagram in Fig. 4c and d, it is shown that the band gap of Co-2MI-G was 0.14 eV, while that of Co-2MI-G + 2NH_3_ was 0.50 eV. It is well explained that Co-2MI-G + 2NH_3_ had stronger insulating properties than Co-2MI-G. Next, we explored the mechanism of the change in conductivity (Fig. 4c and d). It is proved that the DOS at a 0.2–0.4 eV range of Co-2MI-G was composed of the coupling of 2p orbitals from carbon and oxygen. However, in the band structure of Co-2MI-G + 2NH_3_, the coupling structure disappeared. As demonstrated in Fig. 4e, the C 2p and O 2p of Co-2MI-G formed an inter-state, making the band gap of Co-2MI-G 0.14 eV. Co single atoms attained a higher oxidation state by bonding with NH_3_, thus leading to the passivated inter-states, widening the band gap from 0.14 eV to 0.50 eV (Fig. 4e). Therefore, the high oxidation state of Co single atoms resulted in the change of conductivity of Co-2MI-G, contributing to enhanced sensing performance.

## CONCLUSION

In summary, we demonstrated a brand-new gas sensor based on the unsaturated coordination chemistry mechanism for sensitive and ultra-selective NH_3_ detection. Co-2MI-G exhibited a resistance response of 67.598% for 1 ppm NH_3_, which outperforms the reported NH_3_ sensors, accompanied by ultrahigh selectivity for 0.5 ppm NH_3_/50 ppm VOCs. *In-situ* characterization techniques confirmed the unique coordination mechanism of single Co atoms towards the adsorbed NH_3_ molecules, involving the upshift of valence state from Co^2+^ to Co^3+^ during NH_3_ exposure and showing robust reversibility. Theoretical calculations revealed that the Co single atom in Co-2MI-G showed a +1.53 Bader charge after adsorbing NH_3_, and passivated the interband states of C 2p and O 2p, resulting in a widened band gap from 0.14 eV to 0.50 eV and then the drastic change in resistance. Therefore, this work will shed light on the fundamentals of the coordination chemistry mechanism and aid in the precise design of single-atom sensors for ultra-selective gas detection at the atomic level.

## METHODS

### Chemicals

Commercial Co(NO_3_)_2_·6H_2_O and 2MI were purchased from Aladdin Co., Ltd. The commercial products were directly used without any treatment. GO solution (6.1 mg mL^−1^) was synthesized by the modified Hummers’ method.

### Preparation of Co-2MI-G

Firstly, 10 mg Co(NO_3_)_2_·6H_2_O and 30 mg 2MI were added into 1 mL H_2_O to form solution A. Secondly, 5 mL GO solution (6.1 mg mL^−1^) was mixed with 25 mL deionized water to obtain solution B. Solution A was poured into solution B under vigorous stirring. After 5 min, the mixed solution was poured into liquid nitrogen to freeze quickly, and subsequently dehydrated to attain the solids by freeze-drying for 2 days. Afterwards, the freeze-dried sample was heated to 100°C for 24 h in a vacuum oven. Finally, Co-2MI-G was acquired by annealing the dried sample at 200°C for 3 h with 10% H_2_/90% Ar protection.

### Fabrication and measurement of the gas sensor

Co-2MI-G was dispersed in ethanol with a concentration of 1 mg mL^−1^, and then sonicated for several minutes to obtain uniform suspension. Subsequently, 20 μL of the slurry was drop-coated on a ceramic tube with two Au electrodes printed on it and connected with two wires. Gas sensing measurements were performed in an 18 L home-made seal chamber equipped with a heating block to evaporate ammonium hydroxide. When the baseline of resistance in air (*R*_0_) was steady, a certain amount of ammonium hydroxide was injected into the heating block in the testing chamber to completely form the vapor. The reaction time of the target gas was fixed at 5 min. After 5 min, the sensor was refreshed with clean air and the resistance recovered back. Gas response was defined as Δ*R*/*R*_0_ = (*R*-*R*_0_)/*R*_0_ × 100%, where *R* and *R*_0_ were the real-time and initial resistances, respectively.

## Supplementary Material

nwaf031_Supplemental_File
