# Supplementary material for: Single cobalt atoms with unconventional dynamic coordination mechanism for selective ammonia sensor
Source: Natl Sci Rev. 2025 Feb 4;12(3):nwaf031. doi: 10.1093/nsr/nwaf031 (PMC11837343; doi:10.1093/nsr/nwaf031)
Supplement: nwaf031_Supplemental_File [file nwaf031_supplemental_file.pdf]

Supporting Information

Single Cobalt Atoms with Unconventional Dynamic Coordination Mechanism for Selective Ammonia Sensor

Yuejiao Li,[1,†] Yaguang Li,[1,3,†] Yushu Shi,[2,4,†] Jianmei Gao,[2] Jianmin Lu,[1] Chao Wang,[1] Junyu Chang,[2] Zhenming Wang,[2,6] Yangyue Yang[2,4] Bing Yang,[5] Liang Feng,[2,] Qiang Fu,[1] Xinhe Bao,[1] and Zhong-Shuai Wu,[1,5,*]*

[1] State Key Laboratory of Catalysis, Dalian Institute of Chemical Physics, Chinese Academy of Sciences, Dalian 116023, China

[2] Department of Instrumentation and Analytical Chemistry, CAS Key Laboratory of Separation Science for Analytical Chemistry, Dalian Institute of Chemical Physics, Chinese Academy of Sciences, Dalian 116023, China

[3] Research Center for Solar Driven Carbon Neutrality, Engineering Research Center of Zero-carbon Energy Buildings and Measurement Techniques, Ministry of Education, The College of Physics Science and Technology, Institute of Life Science and Green Development, Hebei University, Baoding, 071002, China

[4] University of Chinese Academy of Sciences, 19 A Yuquan Rd, Shijingshan District, Beijing 100049, China

[5] Dalian National Laboratory for Clean Energy, Chinese Academy of Sciences, 457 Zhongshan Road, Dalian 116023, China

[6] Environmental Research Institute, Shandong University, Qingdao 266237, China

∗Corresponding author. E-mail: fengl@dicp.ac.cn (L. Feng); wuzs@dicp.ac.cn (Z.-S. Wu);

[†] Equally contributed to this work.

1. Materials and methods

Preparation of graphene: 5 mL GO solution (6.1 mg mL^{-1}) was mixed with 25 mL deionized water. After stirring for 5 min, the solution was poured into liquid nitrogen to be freezed quickly, and then dehydrated to form the solids by freeze-drying for 2 days. Finally, the graphene (G) was acquired by annealing the freeze-dried sample at 200 ℃ for 3 h with 10% H_2/90% Ar protection.

Preparation of Co-O-G: The synthesis of Co-O-G was similar to that of Co-2MI-G except the preparation of solution A and the final annealing process. In this case, solution A was the mixture of 1 mg $Co(NO_3)_2$ $6H_2O$ and 1 mL H_2O. And the annealing was conducted at 400 ℃ for 3 h with Ar protection.

Preparation of Co-N-G: The synthesis of Co-N-G was similar to that of Co-2MI-G except the preparation of solution A and final annealing process. In this case, solution A was the mixture of 1 mg $Co(NO_3)_2$ $6H_2O$ and 30 mg 2MI added into 1 mL H_2O. And the final annealing was done at 400 ℃ for 3 h with Ar protection.

Preparation of CoO-G: The synthesis of CoO-G was similar to that of Co-2MI-G and the two differences were the solution A and final annealing process. In this case, solution A was the mixture of 30 mg $Co(NO_3)_2$ $6H_2O$ and 1 mL H_2O. And the final annealing process was performed at 400 ℃ for 3 h with Ar protection.

Preparation of 2MI-G: The synthesis of 2MI-G was similar to that of Co-2MI-G and the only difference was the solution A. In this case, solution A was the 30 mg 2MI added into 1 mL H_2O.

Preparation of Co-2MI-G with different ratios: The synthesis of Co-2MI-G with different ratios was similar to that of Co-2MI-G and the only difference was the mass of $Co(NO_3)_2$ $6H_2O$ and 2MI.

Co-2MI-G-1: 5 mg $Co(NO_3)_2$ $6H_2O$ and 15 mg 2MI.

Co-2MI-G-3: 15 mg $Co(NO_3)_2$ $6H_2O$ and 45 mg 2MI.

2. Characterization methods

The crystal structure, morphology and element information of samples were characterized by powder X-ray diffraction (XRD, D2 PHASER, BRUKE, Germany), scanning electron microscopy (JSM-7900F), transmission electron microscopy (TEM, JEM-2100), atomic force microscopy (AFM, Cypher ES), Raman spectroscopy (LabRAM HR 800, with a laser source of 532 nm), and X-ray photoelectron spectroscopy (XPS, Thermo ESCALAB-250). The aberration-corrected high-angle annular dark-field (HAADF) scanning transmission electron microscopy (STEM) was detected by ARM 200F, equipped with spherical aberration corrector. Micromeritics Tristar 3020 system was used for obtaining specific

surface area and pore size distribution of samples. The near Co K-edge X-ray absorption spectroscopy (XANES) and extended X-ray absorption fine structure (EXAFS) data were tested by the BL14W1 beamline of Shanghai Synchrotron Radiation Facility (SSRF). Athena software was used to calibrate the energy scale, correct the background of signals, and normalize intensity of signals and Fourier transform the data from *k*-space to *r*-space. Fourier transform infrared (FTIR) spectrum was performed to characterize the surface functional groups of Co-2MI-G. Quasi in-situ XPS characterizations were used to analyze the composition of the Co-2MI-G during NH_3 exposing and evaporation. All the tests were carried out at a base pressure of 10^{-9} Torr, which typically required a strong evacuation of 1 h. Electron paramagnetic resonance (EPR) spectra were used to analyze the unpaired electrons during NH_3 sensing, performed in a paramagnetic tube at room temperature. The NH_3 atmosphere of quasi in-situ XPS and EPR was obtained by bubbling $NH_3 \cdot H_2O$ with air at a pressure of 1 atm, and NH_3 evaporation was volatilized by slightly heating to 100 ℃.

3. First principle calculations

We employed the Vienna Ab Initio Package (VASP)[1, 2] to perform the density functional theory (DFT) calculations within the generalized gradient approximation (GGA) using the PBE formulation.[3] We chose the projected augmented wave (PAW) potentials to describe the ionic cores and take valence electrons into account using a plane wave basis set with a kinetic energy cutoff of 400 eV. Partial occupancies of the Kohn−Sham orbitals were allowed using the Gaussian smearing method and a width of 0.05 eV. The electronic energy was considered self-consistent when the energy change was smaller than 10^{-5} eV. A geometry optimization was considered convergent when the force change was smaller than 0.02 eV/Å. Grimme's DFT-D3 methodology[4] was used to describe the dispersion interactions.

The equilibrium lattice constant of hexagonal graphene unit cell separated by a vacuum layer in the depth of 15 Å was optimized, when using a 15 × 15 × 1 Monkhorst-Pack k-point grid for Brillouin zone sampling, to be a = 2.468 Å. We then used it to construct a graphene sheet model with *p*(7x7) periodicity in the *x* and *y* directions and 1 atomic layer in the *z* direction by vacuum depth of 15 Å in order to separate the surface slab from its periodic duplicates. This graphene sheet model contains 98 C atoms. During structural optimizations, the gamma point in the Brillouin zone was used for k-point sampling, and all atoms were allowed to relax.

The adsorption energy (E_{ads}) of adsorbate A was defined as $E_{ads} = E_{A/surf} - E_{surf} - E_{A(g)}$

where $E_{A/surf}$, E_{surf} and $E_{A(g)}$ are the energy of adsorbate A adsorbed on the surface, the energy of clean surface, and the energy of isolated A molecule in a cubic periodic box with a side length of 20 Å and a 1×1×1 Monkhorst-Pack k-point grid for Brillouin zone sampling, respectively.

4. Results and discussion

Table S1. EXAFS fitting parameters of Co from Co-2MI-G and Co-2MI-G+NH_3 extracted from the Co K-edge. The amplitude reduction factor S_0^2 is 0.735. CN is the coordination number. σ^2 is the Debye–Waller factor to account for both thermal and structural disorders. *R* is the distance between absorber and backscatter atoms. ΔE_0 is the inner potential correction.

Sample	Path	CN	$\sigma^2 (10^{-3}$ Å$^2)$	*R*	ΔE_0 (eV)
Co-2MI-G	Co-O/N	2	9.82	1.91	-0.73
Co-2MI-G+NH_3	Co-O/N	4	3.75	1.99	-1.75

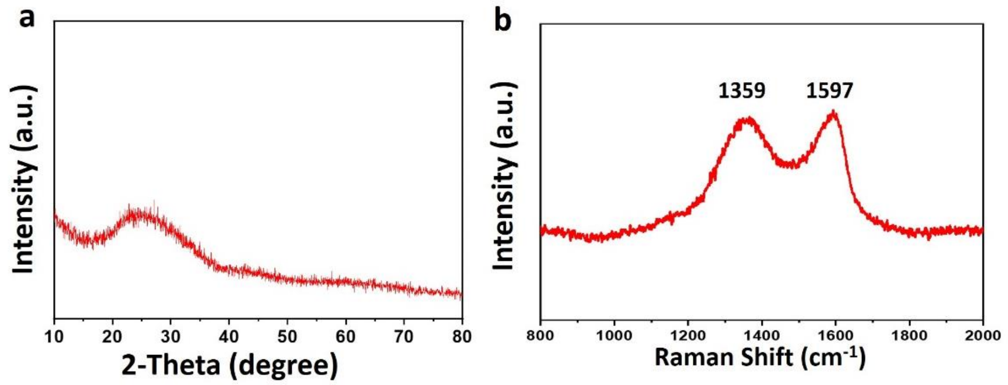

Figure S1. (a) XRD pattern and (b) Raman spectrum of Co-2MI-G.

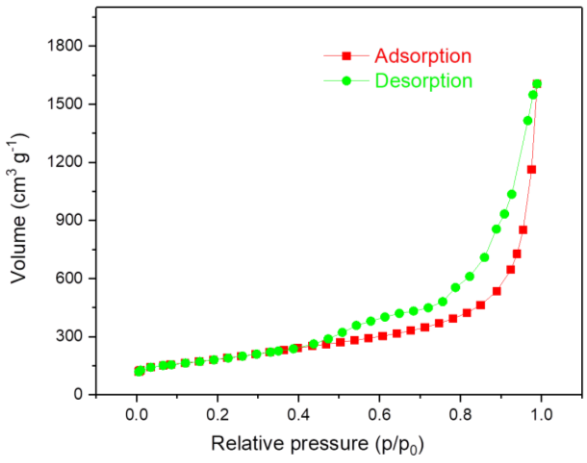

Figure S2. Nitrogen adsorption-desorption isotherm of Co-2MI-G.

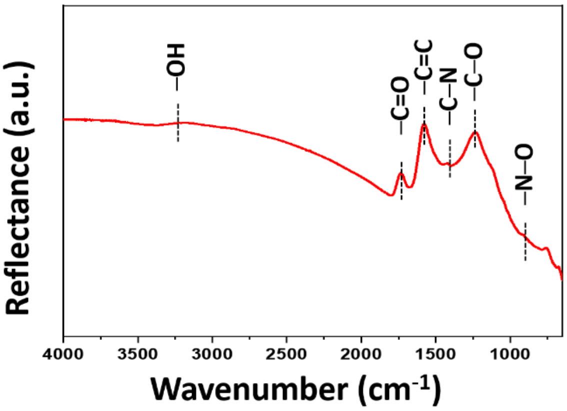

Figure S3. FTIR spectrum of Co-2MI-G.

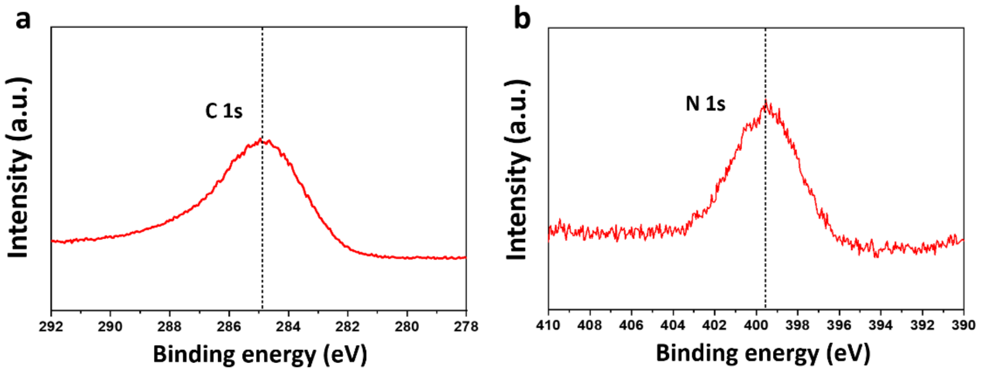

Figure S4. C 1s and N 1s XPS spectra of Co-2MI-G.

To confirm the functional groups of Co-2MI-G, the FTIR analysis was performed, and it can be found that, in addition to the C=C, C–O bonds originating from graphene, C–N bonds appeared, which may be attributed to the imidazole ring **(Figure S3)**.[5] The C 1s and N 1s XPS spectra identify the presence of small molecules 2MI in Co-2MI-G (**Figure S4**).[6] Further, it can be seen that the N in Co-2MI-G does not contain the NO_2 groups with high binding energy (~ 405.98 eV).[7]

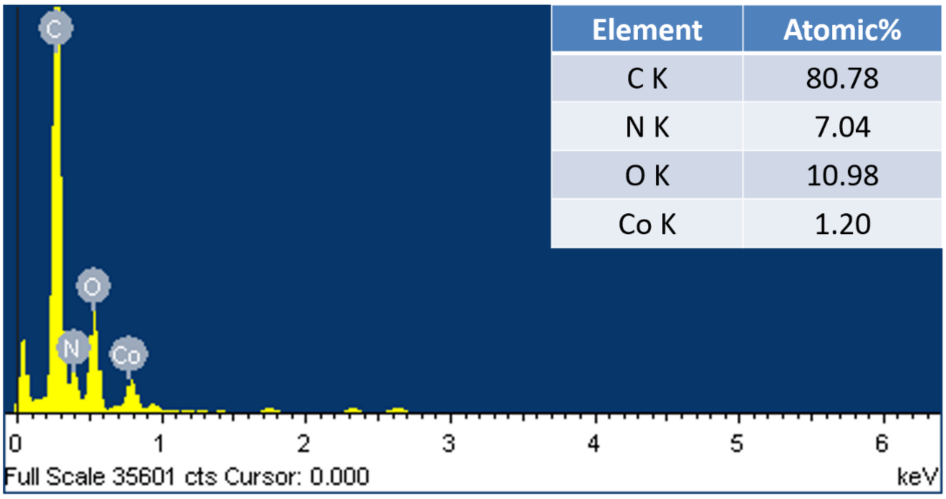

Figure S5. Energy dispersive X-ray spectroscopy (EDS) spectrum of Co-2MI-G.

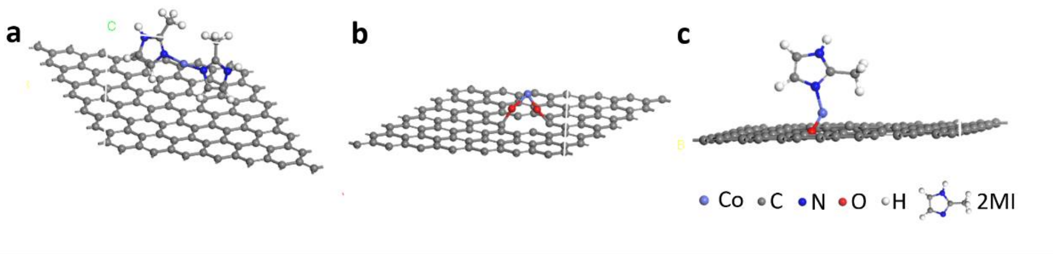

Figure S6. The atomic structures of (a) 2MI-Co-2MI, (b) G-O-Co-O-G, and (c) G-O-Co-2MI. Pink, gray, blue, red, and pale spheres were representative of the Co, C, N, O and H atoms, respectively.

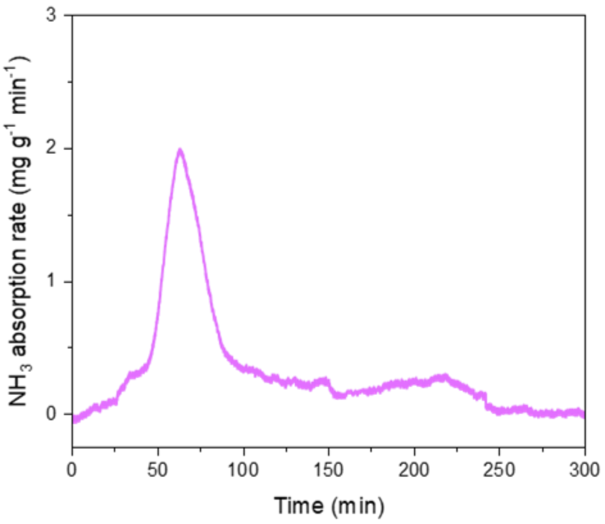

Figure S7. The NH_3 adsorption curve of Co-2MI-G at room temperature.

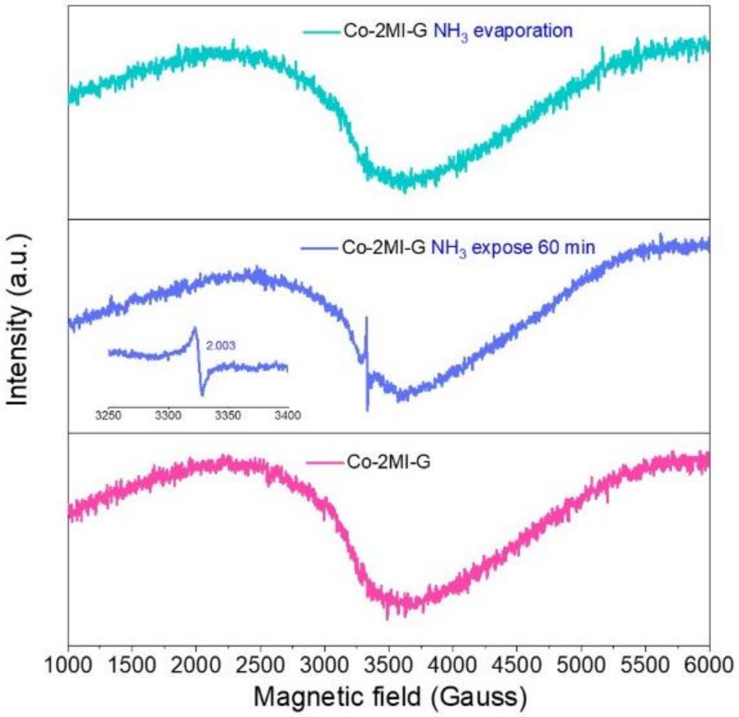

Figure S8. *Ex*-situ EPR spectra of the pristine and Co-2MI-G after exposure to NH_3 for 60 min, followed by NH_3 evaporation.

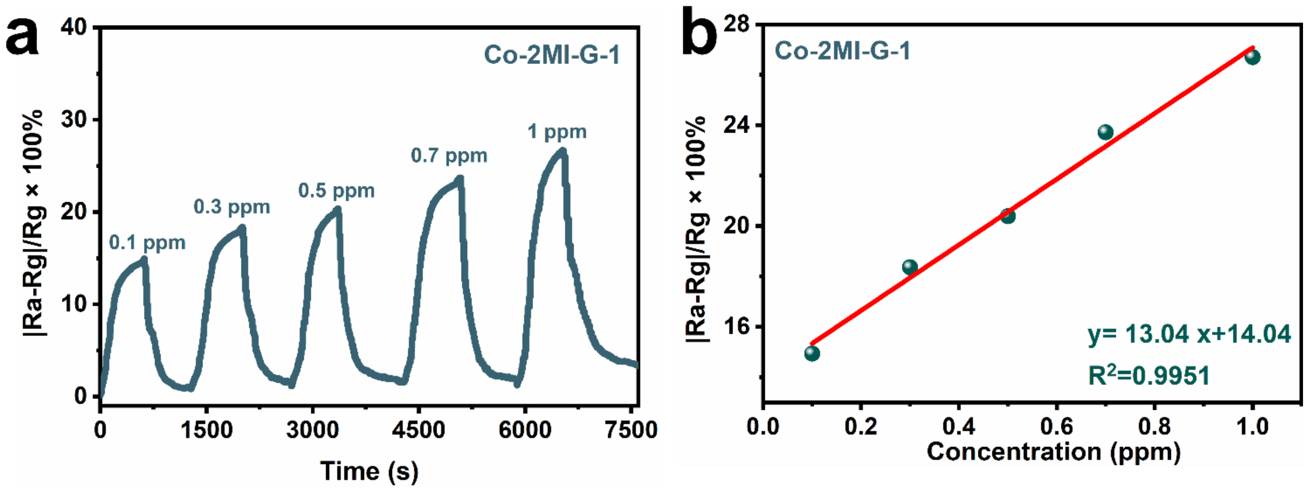

Figure S9. The response curve (a) and linear fitting curve (b) of Co-2MI-G-1.

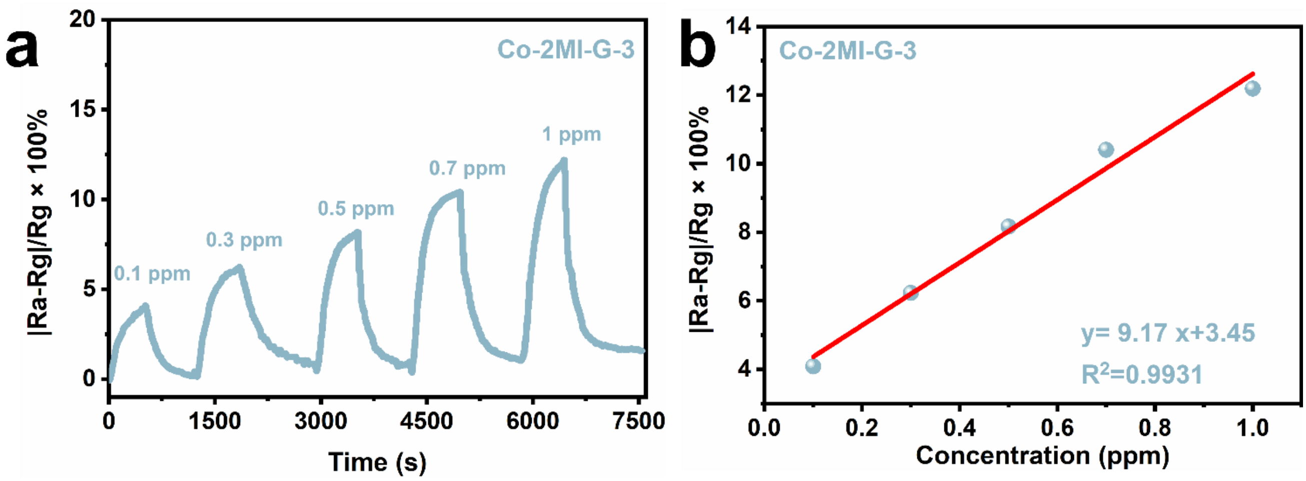

Figure S10. The response curve (a) and linear fitting curve (b) of Co-2MI-G-3.

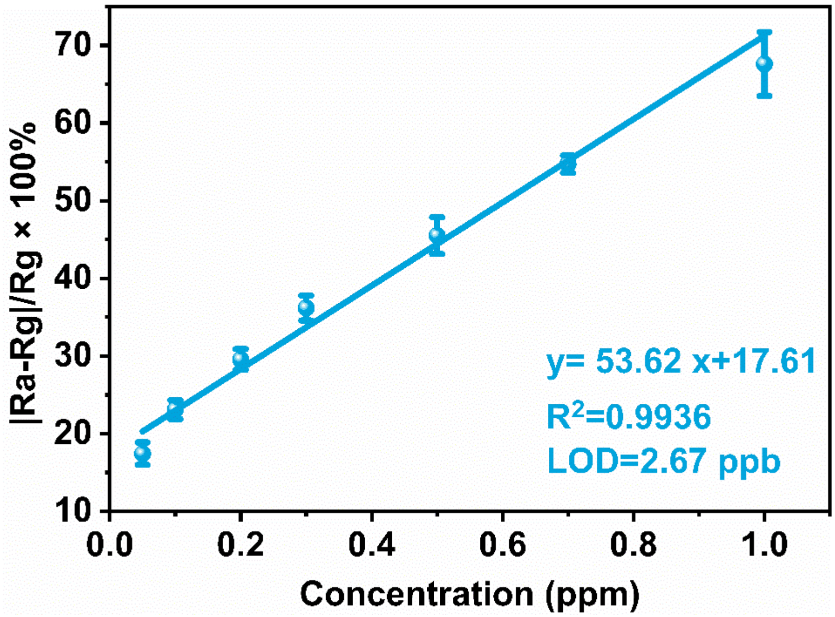

Figure S11. The linear relationship between resistance response ($\Delta R/R_0$, %) and concentration of NH_3 from 0.4 to 10 ppm (Co-2MI-G). As shown in **Fig. S11,** the calculated limit of detection (LOD) is 2.67 ppb.

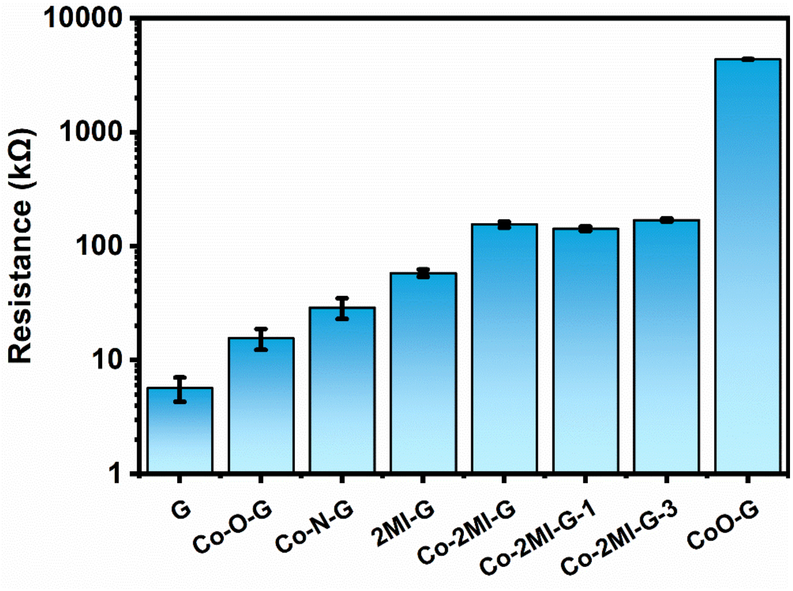

Figure S12. The baseline resistance of different samples.

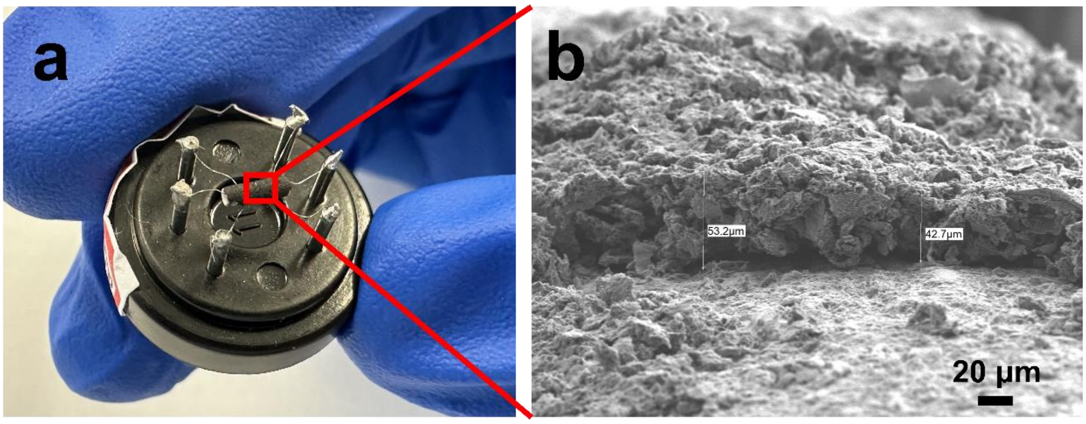

Figure S13. Optical image of physical structure of the gas sensor (a) and SEM image of the thickness of the sensing membrane cross-section (b).

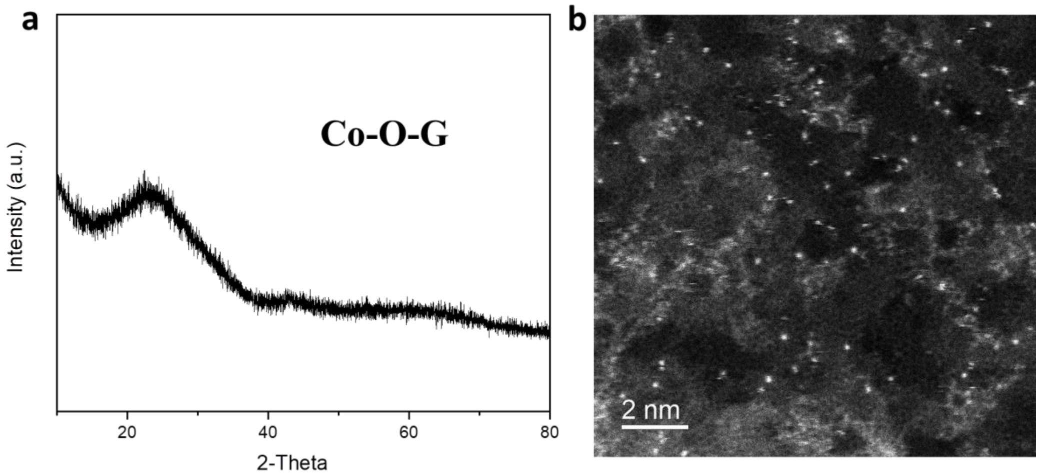

Figure S14. (a) XRD pattern and (b) HAADF-STEM image of Co-O-G.

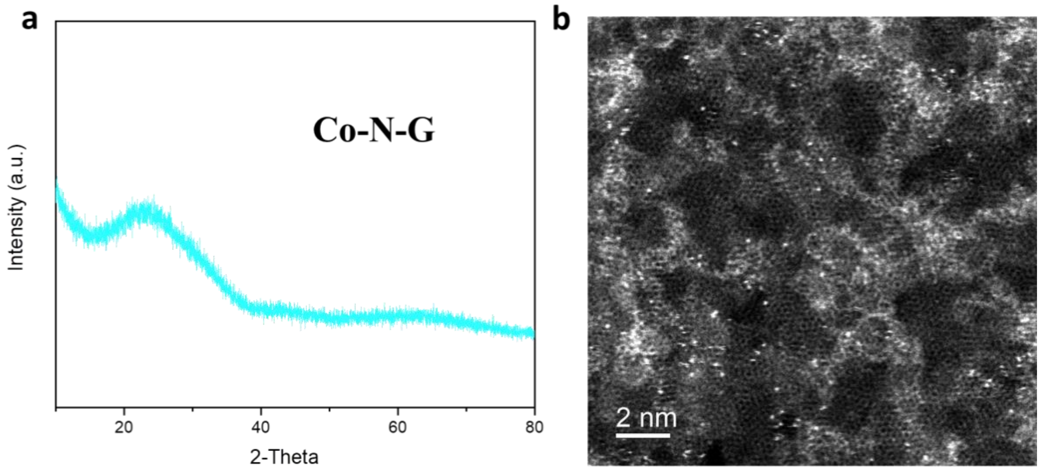

Figure S15. (a) XRD pattern and (b) HAADF-STEM image of Co-N-G.

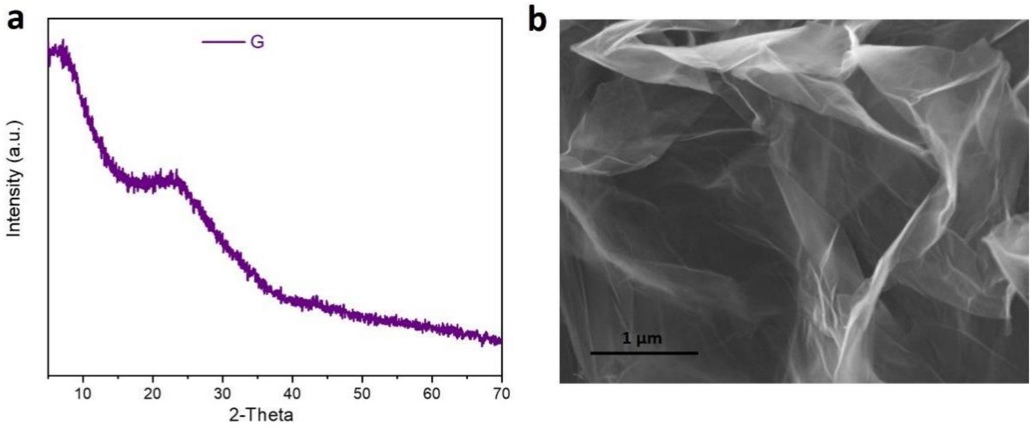

Figure S16. XRD pattern (a) and SEM image (b) of G.

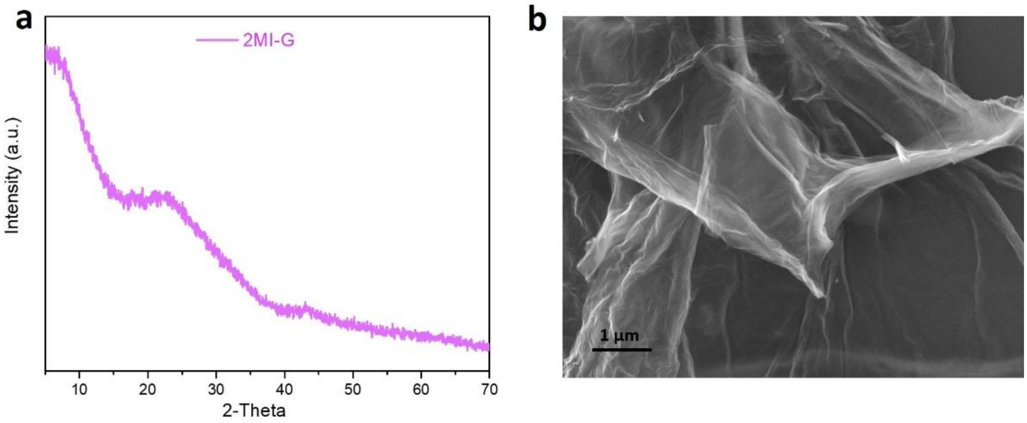

Figure S17. XRD pattern (a) and SEM image (b) of 2MI-G.

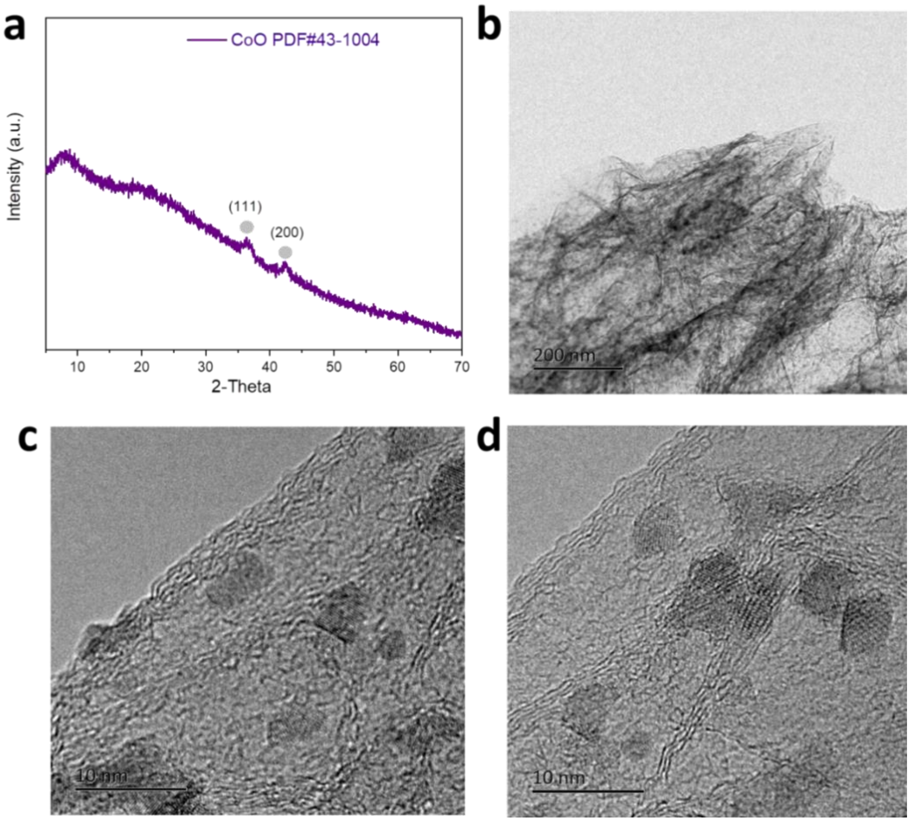

Figure S18. (a) XRD pattern, (b) TEM image, and (c, d) HRTEM images of CoO-G.

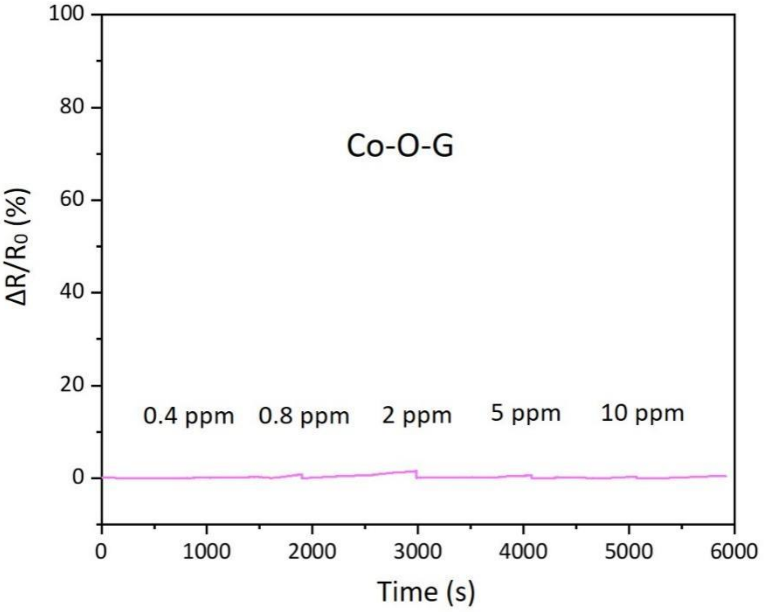

Figure S19. The resistance response curve of Co-O-G to NH_3 from 0.4 to 10 ppm.

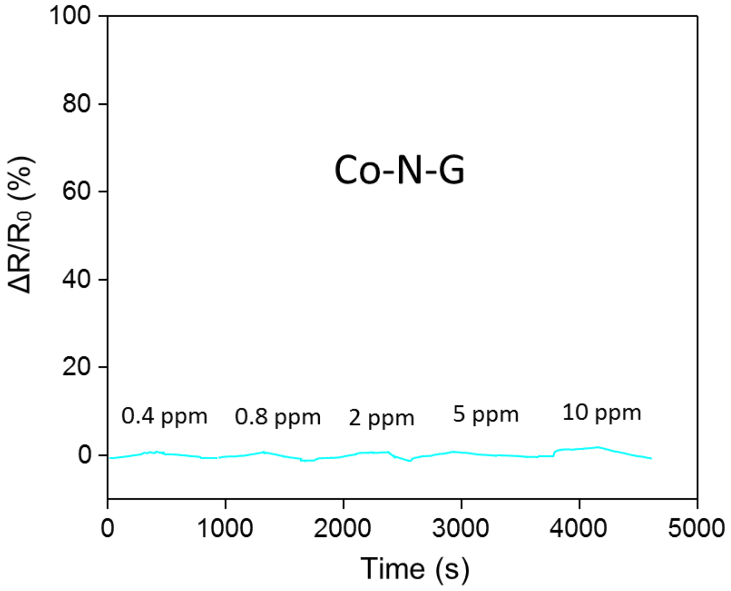

Figure S20. The resistance response curve of Co-N-G to NH_3 from 0.4 to 10 ppm.

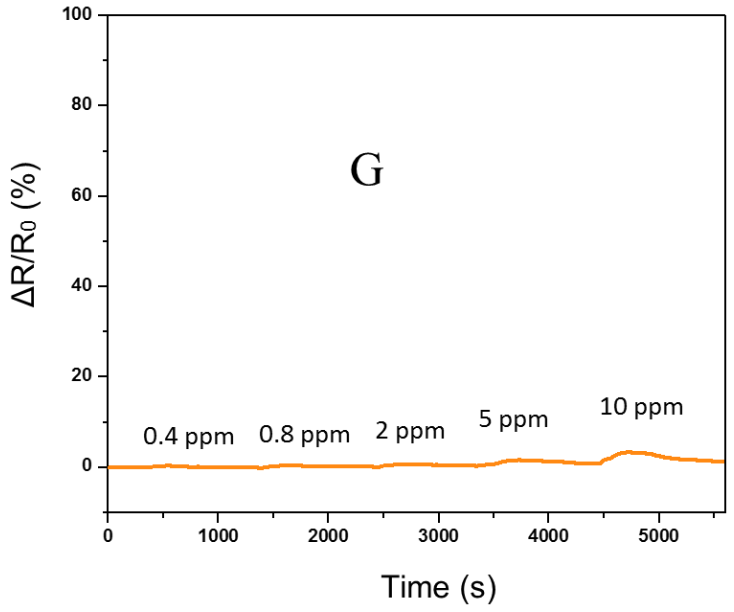

Figure S21. The resistance response curve of G to NH_3 from 0.4 to 10 ppm.

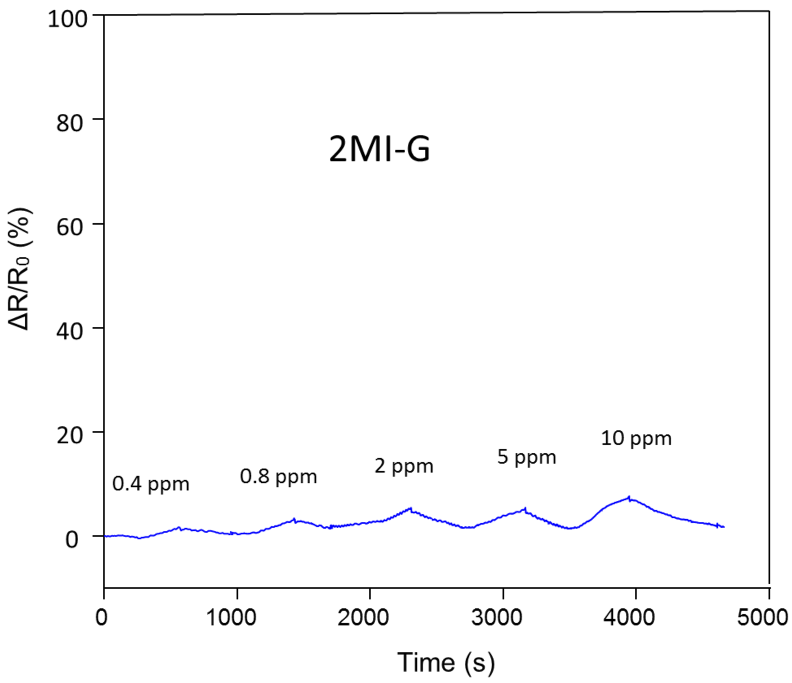

Figure S22. The resistance response curve of 2MI-G to NH_3 from 0.4 to 10 ppm.

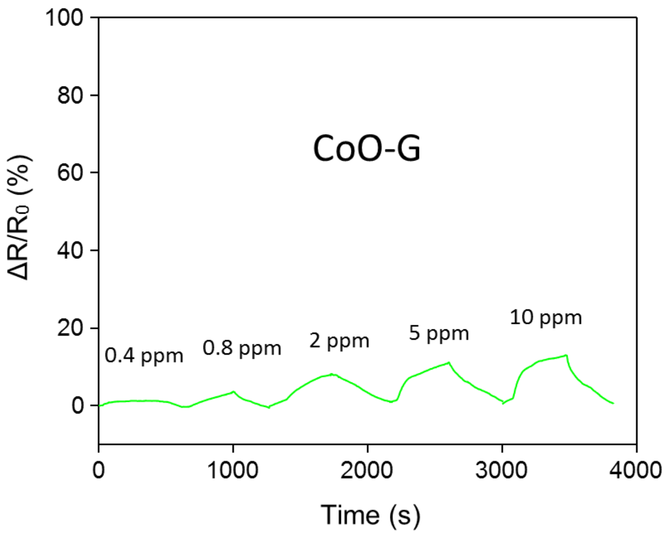

Figure S23. The resistance response curve of CoO-G to NH_3 from 0.4 to 10 ppm.

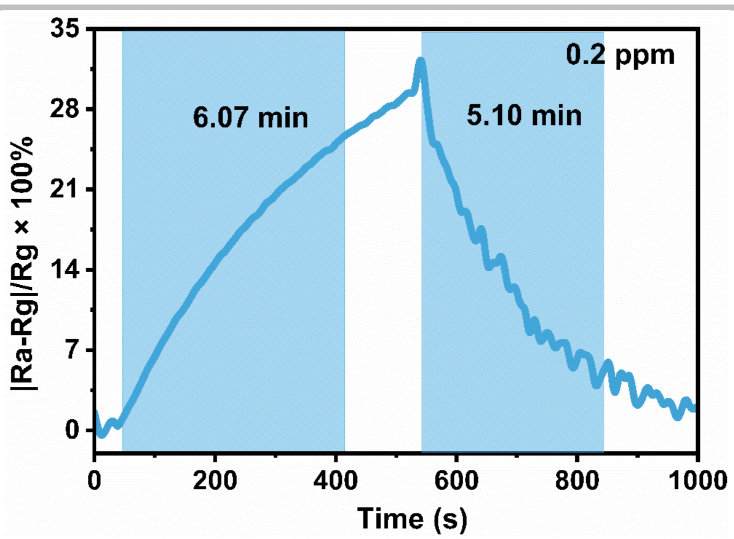

Figure S24. The response recovery time of the Co-2MI-G.

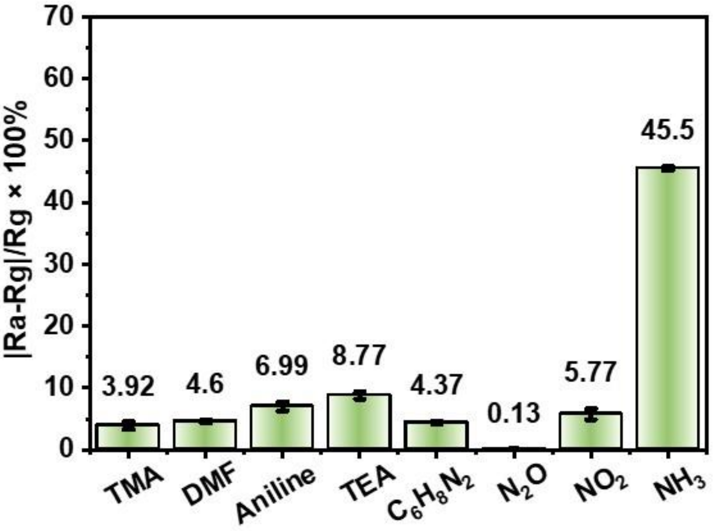

Figure S25. The sensing response of Co-2MI-G towards 0.5 ppm of NH_3, 50 ppm trimethylamine (TMA), N,N-dimethylformamide (DMF), aniline, triethylamine (TEA), 2,5-dimethylpyrazine ($C_6H_8N_2$), N_2O, and NO_2.

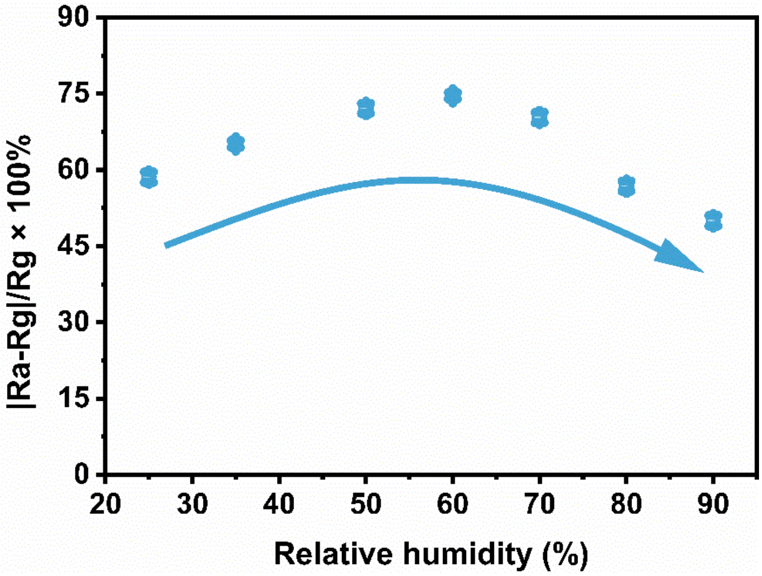

Figure S26. The response of Co-2MI-G to 5 ppm NH_3 under different humidity from 25% to 90%.

It was found that with the increase of humidity, the sensor's response exhibited an initial increasing followed by a subsequent decreasing trend. This phenomenon can be attributed to the high solubility of NH_3 in H_2O. Specifically, an appropriate addition of water adsorbed on the surface of sensor material can result in an enrichment effect on trace NH_3, thereby improving the sensor performance. Conversely, excessive water leads to a significant amount of H_2O occupying the material surface, which masks the adsorption and reaction sites, consequently reducing the sensor performance.

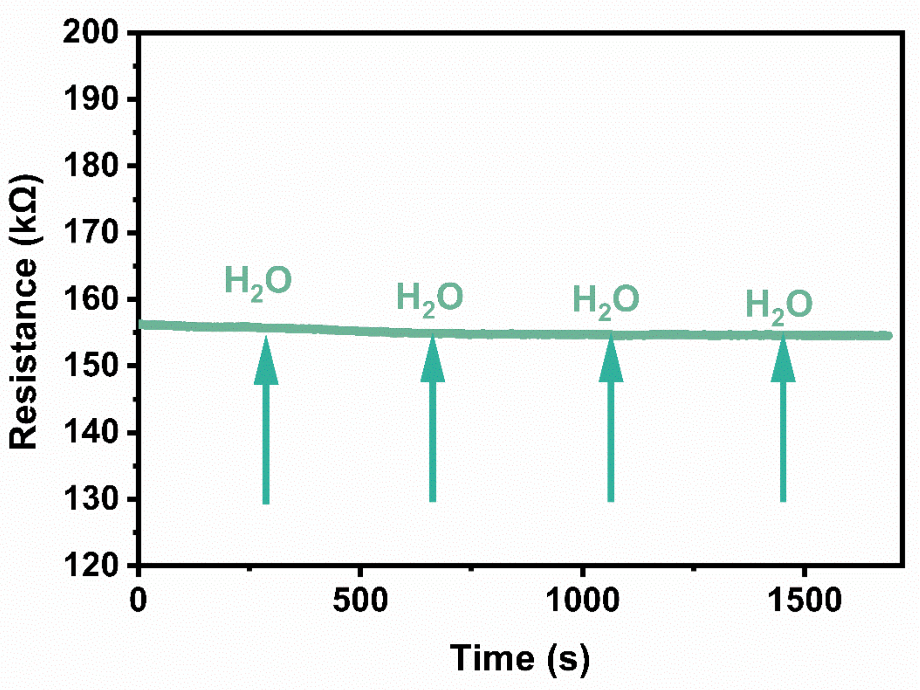

Figure S27. The impact of H_2O on sensor resistance, the concentration of H_2O is 1000 ppm.

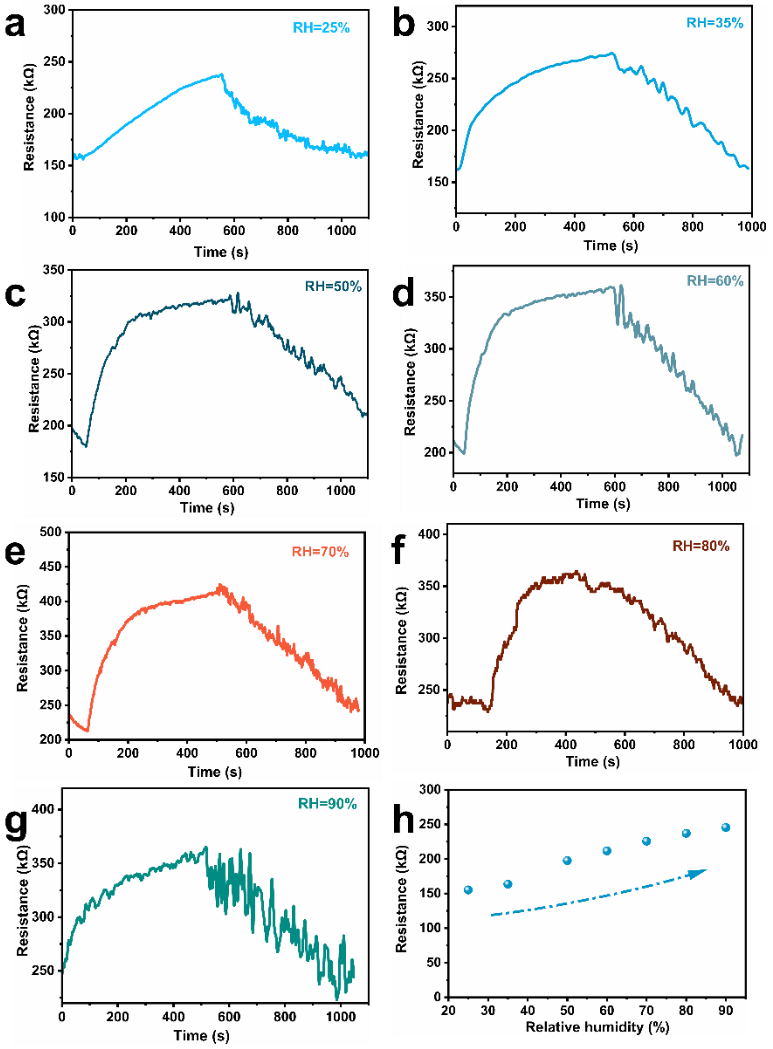

Figure S28. The real-time resistance changes of the sensor under different humidity levels (a-g) and the summary of baseline resistance under different humidity levels (h).

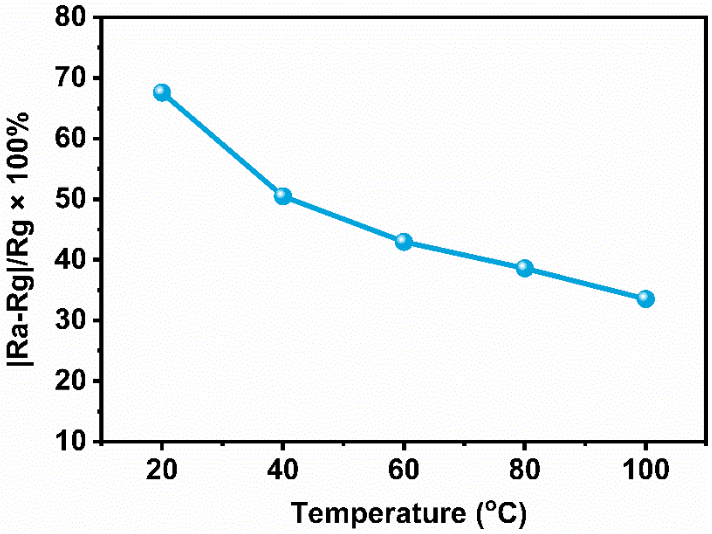

Figure S29. The sensor performance varies with temperature.

The response value of the sensor was observed to decrease at higher temperatures, while the response recovery time was seen to accelerate. The increased temperature facilitates the diffusion of gas molecules, which in turn accelerates the response recovery time. However, an elevated temperature can also exacerbate the instability observed during the coordination of single atoms of N and Co, which could in turn lead to a decrease in the sensor's response value.

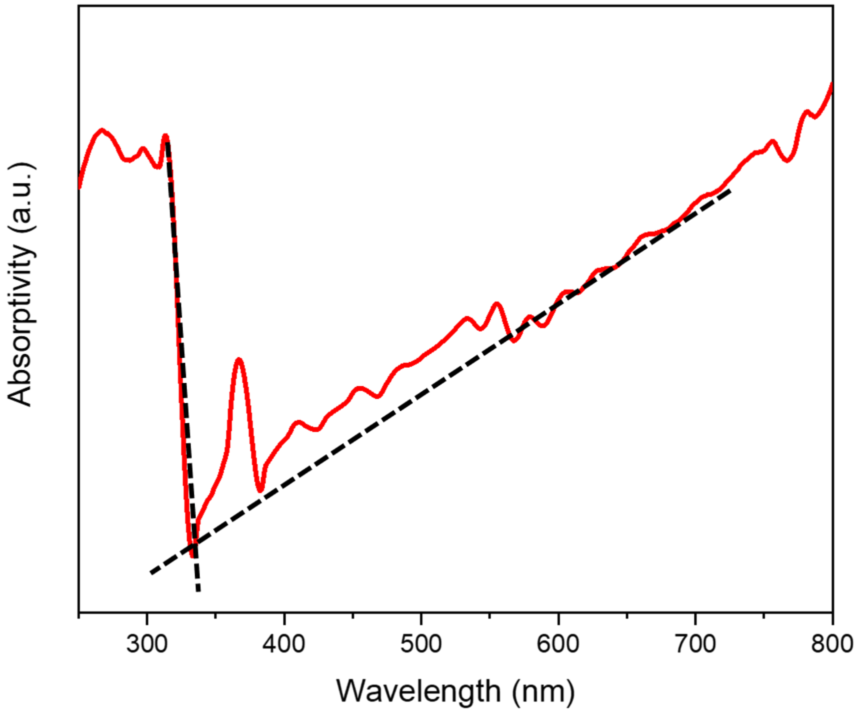

Figure S30. UV-vis spectrum of Co-2MI-G.

The light absorption of Co-2MI-G is originated from the hybridization of Co single atoms, 2MI and graphene. It is speculated that the absorption edge near 330 nm might be caused by the bonding system of Co single atoms and 2MI, while the absorption trail on the visible and infrared light regions might be originated from graphene.

Table S2. A Comparison of our NH_3-sensing results of Co-2MI-G with recently reported sensing materials.

**Materials**	**Gas**	**Concentration (ppm)**	**Temperature (°C)**	**Response**	**Refs.**
Co-2MI-G	NH_3	1	RT	67.598%	This work
ZnO/RGO	NH_3	50	RT	19.2%	[8]
SiO_2-CRGO	NH_3	50	RT	31.5%	[9]
WS_2/CuO	NH_3	10	RT	76.1%	[10]
MXene/CuO	NH_3	100	RT	100%	[11]
ME-Ni-N-C/$Ti_3C_2T_x$	NH_3	5	RT	33.2%	[12]
Ni/graphene	NH_3	50	RT	6.6%	[13]
SnO_2 Nanosheets	NH_3	100	RT	106.5%	[14]
CA/PEGDA	NH_3	20	RT	52%	[15]
Cu-MOF	NH_3	10	RT	~ 45%	[16]
DM-PG	NH_3	10	RT	42%	[17]
w-mPPy@GO	NH_3	10	RT	42%	[18]
Py-GO/PANI	NH_3	10	RT	~ 25%	[19]
NiPc-Ni MOF	NH_3	10	RT	~ 22%	[20]
PCVA Hydrogel	NH_3	10	RT	~ 5%	[21]
3D Graphene Hydrogel	NH_3	10	RT	5%	[22]

Table S3. An overview of the response and recovery times of the sensors at room temperature (RT).

Materials	Operating condition	Response time (s)	Recovery time (s)	Refs.
Co-2MI-G	RT	6.07 min	5.10 min	This work
Pd_1-cMOF	Dry air, RT	4.1 min at 3 ppm	11.1 min at 3 ppm	[23]
$Bi_xIn_{2-x}O_3$ (x = 0.04)	Under blue light, RT	/	10.1 min at 1 ppm	[24]
$Fe_2Mo_3O_8$/ MoO_2@MoS_2	5% RH, RT	~ 3 min	60 min	[25]
Conductive Polymer/MOF Films	RT	~ 10 min	~ 15 min	[26]
MMA-90	RT	~ 150 min	~ 100 min	[27]

6. References

1. Kresse G, Furthmuller J. Efficiency of ab-initio total energy calculations for metals and semiconductors using a plane-wave basis set. *Comput Mater Sci* 1996; **6**: 15-50.
2. Kresse G, Furthmuller J. Efficient iterative schemes for ab initio total-energy calculations using a plane-wave basis set. *Phys Rev B* 1996; **54**: 11169-86.
3. Perdew JP, Burke K, Ernzerhof M. Generalized gradient approximation made simple. *Phys Rev Lett* 1996; **77**: 3865-8.
4. Grimme S, Antony J, Ehrlich S *et al.* A consistent and accurate ab initio parametrization of density functional dispersion correction (DFT-D) for the 94 elements H-Pu. *J Chem Phys* 2010; **132**(15): 154104.
5. Wu C, Liu Q, Chen R *et al.* Fabrication of ZIF-8@SiO_2 micro/nano hierarchical superhydrophobic surface on AZ31 magnesium alloy with impressive corrosion resistance and abrasion resistance. *ACS Appl Mater Interfaces* 2017; **9**: 11106-15.
6. Xue G, Dai QP, Jiang SG. Chemical-reactions of imidazole with metallic silver studied by the use of SERS and XPS techniques. *J Am Chem Soc* 1988; **110**: 2393-5.
7. Holzinger M. Characterization of oxidized SWCNTs by XPS. *AIP Conference Proceedings* 2002; **633**(1): 96-9.
8. Wang T, Sun Z, Huang D *et al.* Studies on NH_3 gas sensing by zinc oxide nanowire-reduced graphene oxide nanocomposites. *Sens Actuators B Chem* 2017; **252**: 284-94.
9. Huang D, Li X, Wang S *et al.* Three-dimensional chemically reduced graphene oxide templated by silica spheres for ammonia sensing. *Sens Actuators B Chem* 2017; **252**: 956-64.
10. Luo H, Shi J, Liu C *et al.* Design of p–p heterojunctions based on CuO decorated WS_2 nanosheets for sensitive NH_3 gas sensing at room temperature. *Nanotechnology* 2021; **32**: 445502.
11. Wang D, Zhang D, Yang Y *et al.* Multifunctional latex/polytetrafluoroethylene-based triboelectric nanogenerator for self-powered organ-like MXene/metal–organic framework-derived CuO nanohybrid ammonia sensor. *ACS Nano* 2021; **15**(2): 2911-9.
12. Quan W, Shi J, Zeng M *et al.* Highly sensitive ammonia gas sensors at room temperature based on the catalytic mechanism of N, C coordinated Ni single-atom active center. *Nano-Micro Lett* 2024; **16**(1): 277.
13. Kim S, Kim Y, Kim J *et al.* Highly selective ammonia detection in NiO-functionalized graphene micropatterns for beef quality monitoring. *Adv Funct Mater* 2024; **34**: 2407885.
14. Verma M, Bahuguna G, Singh S *et al.* Porous SnO_2 nanosheets for room temperature ammonia sensing in extreme humidity. *Mater Horiz* 2024; **11**(1): 184-95.

15. Liu L, Fei T, Guan X *et al.* Highly sensitive and chemically stable NH_3 sensors based on an organic acid-sensitized cross-linked hydrogel for exhaled breath analysis. *Biosens Bioelectron* 2021; **191**:113459.
16. Yao M-S, Lv X-J, Fu Z-H *et al.* Layer-by-layer assembled conductive metal–organic framework nanofilms for room-temperature chemiresistive sensing. *Angew Chem Int Ed* 2017; **56**: 16510-4.
17. Qin J, Gao J, Shi X *et al.* Hierarchical ordered dual-mesoporous polypyrrole/graphene nanosheets as bi-functional active materials for high-performance planar integrated system of micro-supercapacitor and gas sensor. *Adv Funct Mater* 2020; **30**: 1909756.
18. Gao J, Qin J, Chang J *et al.* NH_3 sensor based on 2D wormlike polypyrrole/graphene heterostructures for a self-powered integrated system. *ACS Appl Mater Interfaces* 2020; **12**: 38674-81.
19. Ly TN, Park S. Highly sensitive ammonia sensor for diagnostic purpose using reduced graphene oxide and conductive polymer. *Sci Rep* 2018; **8**: 18030.
20. Meng Z, Aykanat A, Mirica KA. Welding metallophthalocyanines into bimetallic molecular meshes for ultrasensitive, low-power chemiresistive detection of gases. *J Am Chem Soc* 2019; **141**: 2046-53.
21. Zhi H, Gao J, Feng L. Hydrogel-based gas sensors for NO_2 and NH_3. *ACS Sens* 2020; **5**: 772-80.
22. Wu J, Wei Y, Ding H *et al.* Green synthesis of 3D chemically functionalized graphene hydrogel for high-performance NH_3 and NO_2 detection at room temperature. *ACS Appl Mater Interfaces* 2020; **12**: 20623-32.
23. Park C, Shin H, Jeon M *et al.* Single-atom catalysts in conductive metal–organic frameworks: enabling reversible gas sensing at room temperature. *ACS Nano* 2024; **18**(38): 26066-75.
24. Park S, Kim M, Lim Y *et al.* Dual-photosensitizer synergy empowers ambient light photoactivation of indium oxide for high-performance NO_2 sensing. *Adv Mater* 2024; **36**(24): 2313731.
25. Li X, Zeng W, Zhuo S *et al.* Highly sensitive room-temperature detection of ammonia in the breath of kidney disease patients using $Fe_2Mo_3O_8/MoO_2@MoS_2$ nanocomposite gas sensor. *Adv Sci* 2024; **11**(32): 2405942.
26. Roh H, Kim D-H, Cho Y *et al.* Robust chemiresistive behavior in conductive polymer/MOF composites. *Adv Mater* 2024; **36**: 2312382.
27. Kim S, Shin H, Lee J *et al.* Three-dimensional MoS_2/MXene heterostructure aerogel for chemical gas sensors with superior sensitivity and stability. *ACS Nano* 2023; **17**: 19387-97.
